# Extensive biofilm covering on sgraffito wall art: a call for proactive monitoring

**DOI:** 10.3389/fmicb.2025.1664404

**Published:** 2026-01-21

**Authors:** Irit Nir, Anath Sharaby, Hana Barak, Mariela J. Pavan, Lonia R. Friedlander, Victor Multanen, Ariel Kushmaro

**Affiliations:** 1 Avram and Stella Goldstein-Goren Department of Biotechnology Engineering, Ben-Gurion University of the Negev, Beer Sheva, Israel; 2 Ilse Katz Institute for Nanoscale Science and Technology, Ben- Gurion University of the Negev, Beer Sheva, Israel; 3 The Goldman Sonnenfeldt School of Sustainability and Climate Change, Ben-Gurion University of the Negev, Beer Sheva, Israel

**Keywords:** sgraffito, wall art, lime mortar, bio-weathering, next-generation sequencing (NGS)

## Abstract

**Background:**

The study focuses on a black and white sgraffito decoration attached to a cement exterior wall in Kibbutz Yiftach, Israel. Since its creation in 1971, the artwork has experienced weathering processes, resulting in peeling, flaking, and the development of a microbial layer on the wall art decoration. Before its restoration in 2022, this study was initiated, aiming to address three primary questions: What is the composition of the microbial communities? What is the distribution of these microbial communities throughout the wall, and how do they interact with the substrate materials?

**Methods:**

Complementary methods, including mineral analysis, microscopic observations, and molecular techniques, were implemented to answer the study questions.

**Results:**

Five main groups of bacteria (e.g., Cyanobacteria, Actinobacteria, Proteobacteria, Bacteroidota, and Chloroflexi), as well as various types of fungi, were revealed. Nevertheless, although the same phyla were detected across samples, each displayed distinct diversity at the order level. Microscopic observations revealed the attachment of microbial components to both the porous plaster and the rough cement.

**Discussion:**

The study shows a well-developed microbial coating with a localized colonization pattern, underscoring the need for continued long-term monitoring of both the wall-art materials and their environmental conditions to support data-driven conservation.

## 1 Introduction

Sgraffito is a wall art technique commonly used in murals and architectural decoration by applying layers of colored plaster or paint to a surface and then scratching or carving away to reveal the layers underneath (Jagiełło, 2022). The term “sgraffito” comes from the Italian word “sgraffire,” meaning “to scratch” (Aldersey-Williams, 2013). This technique can create a wide range of designs, from simple geometric patterns to more complex, detailed images, and is notable for its ability to add depth and texture to a surface through carved lines and shapes (Baker, 2007). The plaster components are mostly composed of lime mortar, a traditional building material made by mixing lime, sand, and water (Elert et al., 2002). The way lime mortars are made involves heating limestone (CaCO3) to turn it into quicklime (CaO), then mixing it with water to make slaked lime, and finally combining it with materials like sand to form the mortar (Wojcieszak et al., 2024). Over time, lime mortar also absorbs carbon dioxide from the air, contributing to a natural hardening process known as carbonation (Campo et al., 2021). For centuries, masonry construction has relied on its flexibility, breathability, and durability (Ashurst, 1983; Carran et al., 2011; Mundy, 2013). Initially, artists created sgraffito designs in monochrome black and white, utilizing soot to create the dark black tone. The sgraffito technique was popular in Italy during the Renaissance to modernize medieval buildings and express religious views, with examples such as the works of Andrea del Sarto and the Palazzo Pitti in Florence (Ferguson, 2010; Jagiełło, 2022). Throughout the 1970s, Israel’s wall decoration artists predominantly employed this approach. Halperin, a local artist knowledgeable in the European sgraffito technique, devised a regional plaster combination that included sand sourced from Nubian sandstone he gathered in the Negev Desert, Israel. To evaluate the quality of the material he created, he asked local artists to experiment with its application in decorating building walls nationwide. These decorations featured local symbolic motifs derived from the natural environment and biblical narratives. The number of locations using the technology decreased over time, and weathering processes began, causing the structures to disintegrate. Simultaneously, a substantial initiative to document and preserve the works has commenced (Farkash and Tal, 2017).

**FIGURE 1:**
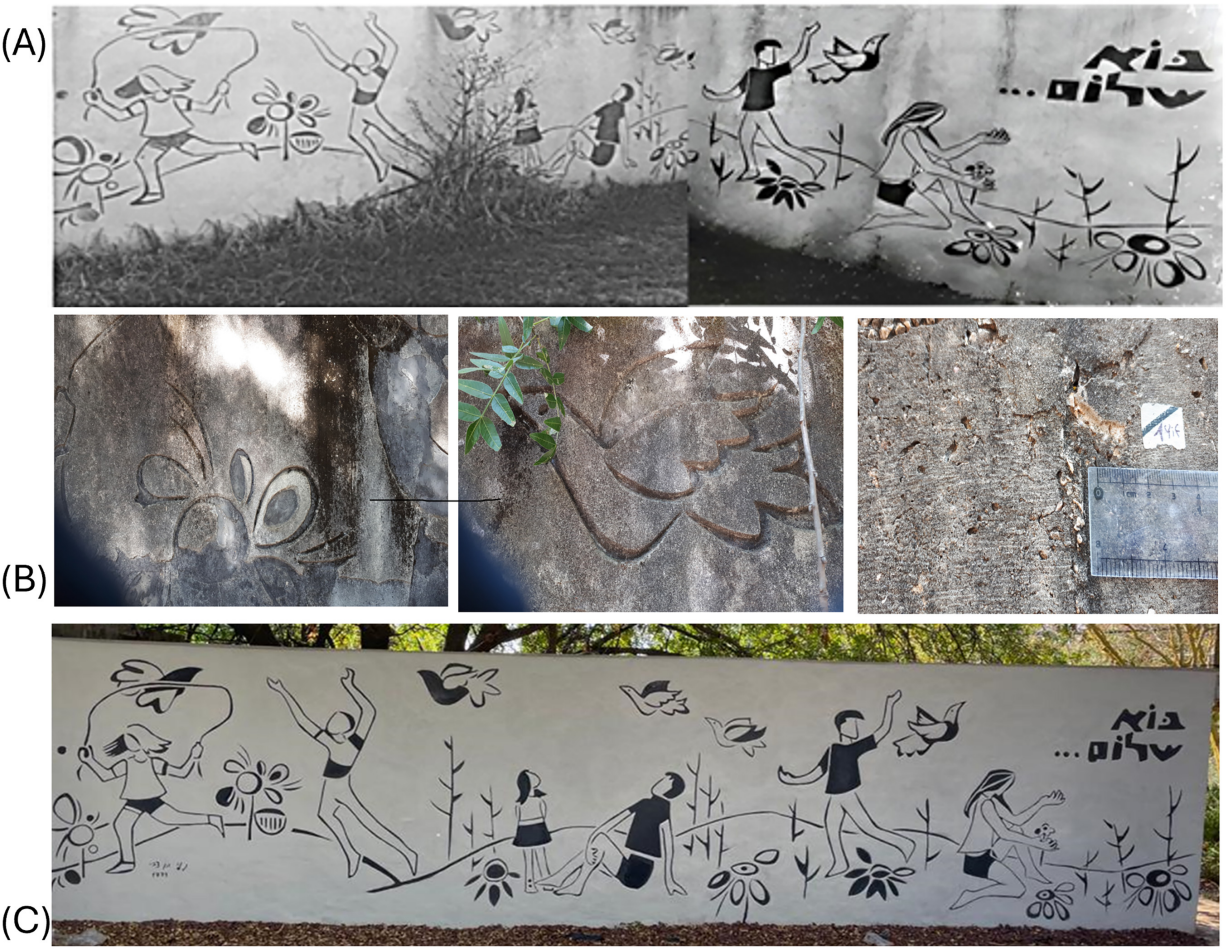
**(A)** The original sgraffito bichromate wall painting titled “Bo-Shalom” (meaning “Come, Peace,” expressing a desire for peace), from its creation period in 1971. **(B)** Deterioration patterns of the wall include dark coating and surface flaking. **(C)** The sgraffito wall art after the cleaning and restoration project (2022).

Sgraffito, like many forms of wall art, is vulnerable to deterioration processes, including biodeterioration. In this process, living organisms such as fungi, bacteria, and algae colonize the surfaces and pores of lime mortar, causing changes to the materials (Cassar, 2002). Microbial growth can lead to staining and discoloration, negatively affecting the aesthetic value of historical and heritage buildings (Gauri and Holdren, 1981; Gu, 2003; Liu et al., 2020; Warscheid and Braams, 2000). In regions with high moisture levels, the plaster and paint layers may absorb water, encouraging the growth of microorganisms. Consequently, sgraffito artworks can experience surface erosion and loss of image details over time (Caron, 2015; Liu et al., 2022). Understanding the mechanisms of microbial colonization and its impact is important when designing conservation strategies for lime mortar structures (McNamara et al., 2010). This study focuses on a sgraffito wall artwork adorning the concrete wall of a shelter in a northern Israeli village called “Kibbutz Yiftach.” Created by a local artist, the piece depicts children playing and dancing in the fields, images that evoke a sense of peace and innocence. To emphasize this contrast and express a desire for peace, the artist inscribed the Hebrew words “Bo Shalom” (“Come Peace”) into the artwork. The artist carved the figurative images through a layer of white plaster, revealing a layer of black plaster underneath (Figure 1A). Since its creation in 1971, the artwork has undergone a weathering process that resulted in a black or dark green cover, loss of aesthetic value, and degradation of the plaster material (Figure 1B). Notwithstanding its symbolic, local communal importance and artistic distinctiveness, the opportunity for cleaning and restoration of the piece did not emerge until 2022 (Figure 1C). Before starting the restoration, this study was initiated, aiming to address three primary questions: What is the composition of the microbial communities? What is their distribution along the wall, and what are their interactions with the substrate materials? The data obtained will expand our knowledge regarding the microbial colonization of sgraffito wall decoration and will serve as a basis for further long-term monitoring and preservation of this unique wall art technique.

## 2 Materials and methods

### 2.1 Study site and sampling

**FIGURE 2:**
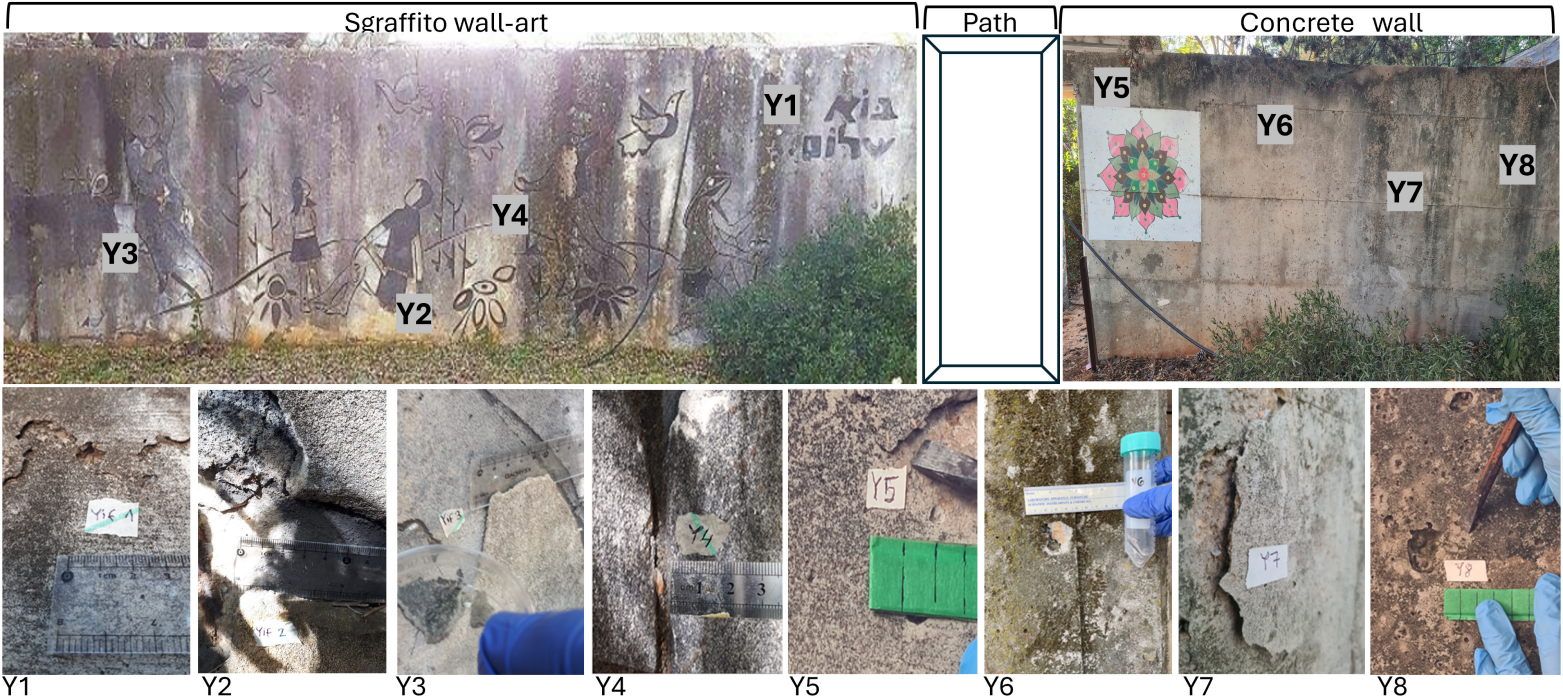
Sampling site: the “Sgraffito” wall art and the adjacent concrete wall, indicating the positions of sampling points Y1-Y8 (above), together with close-up photographs of the samples collected in this study (at the lower portion of the figure).

The sgraffito wall decoration entitled “Bo-shalom” is situated on an external concrete wall, which is a part of a shelter complex adjacent to school buildings in the “Kibbutz Yiftach” village, located in northern Israel (Figure 1). Climatically, the area is classified as a Mediterranean climate zone characterized by an annual precipitation of approximately 530 mm, which primarily occurs during the winter months. July is the hottest month in the region, with an average temperature of about 27°C (81°F), while January is the coldest at about 11°C (52°F). The climatic data were sourced from the Kibbutz Yiftach meteorological station, which is located about 500 m from the study site (33.1259°N, 35.5521°E), and published on the Israel Meteorology Services (IMS) website^1^ (accessed 15.11.2025) (Israel Meteorological Service, 2025) and are presented in Supplementary Table 1. Samples were collected at four points along the wall decoration (Y1–Y4) and at four points along the adjoining concrete wall (Y5–Y8), oriented in the same direction, which were covered by spots of dark green and black crust layer (Figure 2). The Samples were collected about a week before the conservation project was conducted in August 2022. A hammer and chisel, surface-sterilized with 70% ethanol were used to collect slabs of about 4 cm in size. Each of the samples was divided into two fragments. One fragment was used for molecular analysis, and the other for microscopic and chemical study. The samples were transferred to the environmental microbiology lab at Ben Gurion University, Israel, for further analysis. Samples targeted for microscopic and chemical examination were preserved at room temperature, whereas those intended for molecular analysis were kept cold at 4°C until processing (The testing methodologies employed for each sample are specified in Supplementary Table 2).

### 2.2 Wall-art materials characterization

To determine the materials that were used for the creation of the Sgraffito, including the pigments that were used, X-ray powder diffraction (XRD), X-ray fluorescence (XRF) analysis, and Raman spectroscopy were implemented.

#### 2.2.1 X-ray powder diffraction analysis

Phase analysis of the mural samples (Y1-Y4) was performed using the XRD method. The samples were hand-ground using an agate mortar and pestle to the consistency of fine silt as described by Bish and Reynolds (1989). Samples were placed on a bias-cut quartz plate, which gives no diffraction in the normal range for cement or geologic materials, for analysis. The data were gathered using an Empyrean II X-ray powder diffractometer (Malvern Panalytical, Almelo, Netherlands) (Kα radiation, λ = 1.541Ǻ) set to 40 kV and 30 mA and with an attached X’Celerator linear detector. Data was collected in Bragg-Brentano reflectance geometry from 10 to 80° 2θ, with a step size of 0.0334° and a counting time of 50 s per step. Phase identification and Rietveld refinement for phase quantification were both performed using HighScore Plus version 5.1 software, in combination with the ICDD Powder Diffraction File database (PDF-5 +) (version 2024). The average error on the quantitative analysis is ± 0.5 wt%.

#### 2.2.2 X-ray fluorescence measurements

The XRF measurements were performed using an Axios XRF Spectrometer (Malvern Panalytical, Ltd., Almelo, Netherlands). Powder samples were mounted directly into the instrument, and analysis of the emitted elemental fluorescence lines was performed on the data as it was collected using the native Panalytical-provided Omnian XRF analysis software.

#### 2.2.3 Raman spectroscopy analysis

Micro-Raman spectroscopy was used to study the pigments and mineral composition of representative samples (Y2 and Y4 from the mural and Y5–Y8 from the exposed concrete wall). The measurements were taken using a LabRAM HR Evolution Confocal Raman Microscope (Horiba Ltd., Kyoto, Japan), which has a Syncerity CCD detector that is cooled to −60 °C and has a resolution of 1,024 × 256 pixels. 785, 532, and 325 nm lasers were used as excitation sources, with power on the sample ranging from 4 to 80 mW for the 785 nm laser, 1–3.4 mW for the 532 nm laser, and 0.3–0.15 mW for the UV laser. The 10× and 50× LWD objectives were used to focus on the Vis and NIR lasers, and the 40× for the UV laser. The confocal hole was set to 100 and 200 mm, using 600 and 1,800 gr/mm gratings for the visible and UV ranges, respectively. Acquisition time ranged from 1 to 60 s. LabSpec 6, version 6.5.1.24, was implemented to acquire baseline-correct, denoise, and average the spectra using. Identification of the compounds was performed either by comparing their spectra with those obtained from Raman spectral databases (KnowItAll Informatics Systems, 2025, John Wiley and Sons Inc., United States) or by comparing them with Raman spectra in the literature.

### 2.3 Microscopic observations of the biofilm structure

The samples collected from the wall decoration site were initially examined under a light stereomicroscope (SMZ1500, Nikon, Japan) at magnifications of up to 40×, without any specific preparation (Supplementary File 3). This allowed us to gather an overview of the morphological characteristics of the biofilm. Images were captured using a digital camera (Nikon 1J5) connected to the microscope for this purpose. To distinguish between inorganic (e.g., substrate materials) and organic materials (e.g., microbial cells) in the biofilm, a light polarized microscope with polarized light (PPL) and cross-polarized light (XPL) was used (DM1750, Leica Microsystems, Germany). Images were taken using a Canon M6 MaII II digital camera. The Thermo Fisher Verios 460L field-emission scanning electron microscope (FESEM) coupled with Energy-Dispersive X-ray Spectroscopy (EDS) was used to characterize the microbial cells-substrate interface. The samples were prepared as follows: after fixation with 2.5% buffered glutaraldehyde, the samples were subsequently dehydrated using an ascending serial ethanol gradient and immersed in a hexamethyl-disilazane (HMDS)/ethanol gradient solution at concentrations of 25, 50, 75, 90, 95, and 100%. The treated specimens were air-dried and then sputter-coated with a 20-nm layer of gold using an EMITECH K575x sputtering device (Emitech Ltd., United Kingdom).

### 2.4 Taxonomic analysis of the biofilm components using molecular methods

#### 2.4.1 DNA extraction

Substrate samples (Y1, Y2, Y3, and Y4 related to lime mortar; Y5, Y6, Y7, and Y8 related to concrete samples) were crushed in a laboratory hood to obtain small aggregates using sterile pliers. Aggregates (0.5 g) from each sample were transferred into separate bead tubes for DNA extraction. DNA extraction was performed using a MoBio PowerSoil DNA Isolation Kit (MoBio Laboratories, Inc., Carlsbad, CA, United States) following the protocol supplied by the manufacturer. The purity and concentration of DNA samples were determined using a NanoDrop ND-1000 spectrophotometer (NanoDrop Technologies, Wilmington, DE, United States). The extracted DNA was submitted to the Genomics and Microbiome Core Facility at Rush University, United States.

#### 2.4.2 DNA sequencing

For bacterial community analysis, the V3–V4 hypervariable region of the 16S rRNA gene was amplified using the primers 341F (5′-CCTACGGGNGGCWGCAG-3′) and 806R (5′-GACTACHVGGGTATCTAATCC-3′) (Caporaso et al., 2011; Klindworth et al., 2013). To study the fungal community, the internal transcribed spacer (ITS) region was copied using the primers ITS1F (5′-CTTGGTCATTTAGAGGAAGTAA-3′) and ITS2R (5′-GCTGCGTTCTTCATCGATGC-3′) (Gardes and Bruns, 1993; White et al., 1990). Amplicon sequencing was performed on an Illumina MiSeq platform (Illumina Inc., San Diego, CA, United States) with paired-end (2 × 300 bp) sequencing at the Genomics and Microbiome Core Facility at Rush University, United States.

#### 2.4.3 Bioinformatic analysis

The DADA2 pipeline (Callahan et al., 2016) was implemented through QIIME 2 for quality filtering, denoising, chimera removal, and amplicon sequence variant (ASV) identification. Based on quality score profiles, we truncated the forward and reverse reads at positions 250 and 225 bp, respectively. No bases were trimmed from the start of the sequences (–p-trim-left-f 0, –p-trim-left-r 0). The minimum overlap between paired-end reads was set to 15 bp, and pseudo-pooling was employed for ASV inference. Chimeric sequences were identified and removed using the pooled method with a minimum fold parent abundance of 4. The maximum expected error (maxEE) filter threshold was set to 4 for both forward and reverse reads. After denoising, the feature table and representative sequences were summarized. Taxonomy was assigned to ASVs using a naive Bayes classifier trained on the Silva 99% reference database (Bokulich et al., 2018; Quast et al., 2013; Yilmaz et al., 2014). Sequences classified as mitochondria or chloroplasts were filtered out from the final feature table and representative sequences. For the fungal ITS sequences, the DADA2 pipeline was implemented with modified parameters to accommodate the higher variability and quality characteristics of the ITS regions. Forward and reverse reads were truncated at 200 bp, with no bases trimmed from the start of the sequences. The maximum expected error filter threshold was set to 5.0 for both forward and reverse reads, and the minimum quality score truncation threshold (–p-trunc-q) was disabled by setting it to 0. The minimum overlap between paired-end reads was reduced to 10 bp to account for the variable length of the ITS region. ASVs were inferred using the independent pooling method, and the chimera checking step was disabled due to the high variability of the ITS regions. Taxonomy was assigned to the resulting ASVs using a naive Bayes classifier trained on the UNITE database version 10 (99% threshold, dated February 19, 2025) (Nilsson et al., 2019).

#### 2.4.4 Statistical analysis

All the statistical analyses were conducted using QIIME 2 version 2021.4 (Bolyen et al., 2019). Raw sequence data were imported and demultiplexed using QIIME 2 standard procedures. To quantify the differences between the studied microbial groups colonizing the mural (Y1, Y2, and Y4) and the concrete (Y5, Y6, and Y7) substrate samples, Bray-Curtis Principal Coordinate Analysis (PCoA) was implemented along with the PERMANOVA statistical test. Additionally, alpha diversity statistical measures were implemented to describe the diversity within samples.

## 3 Results

### 3.1 Wall-art materials analysis

#### 3.1.1 XRD analysis

**FIGURE 3:**
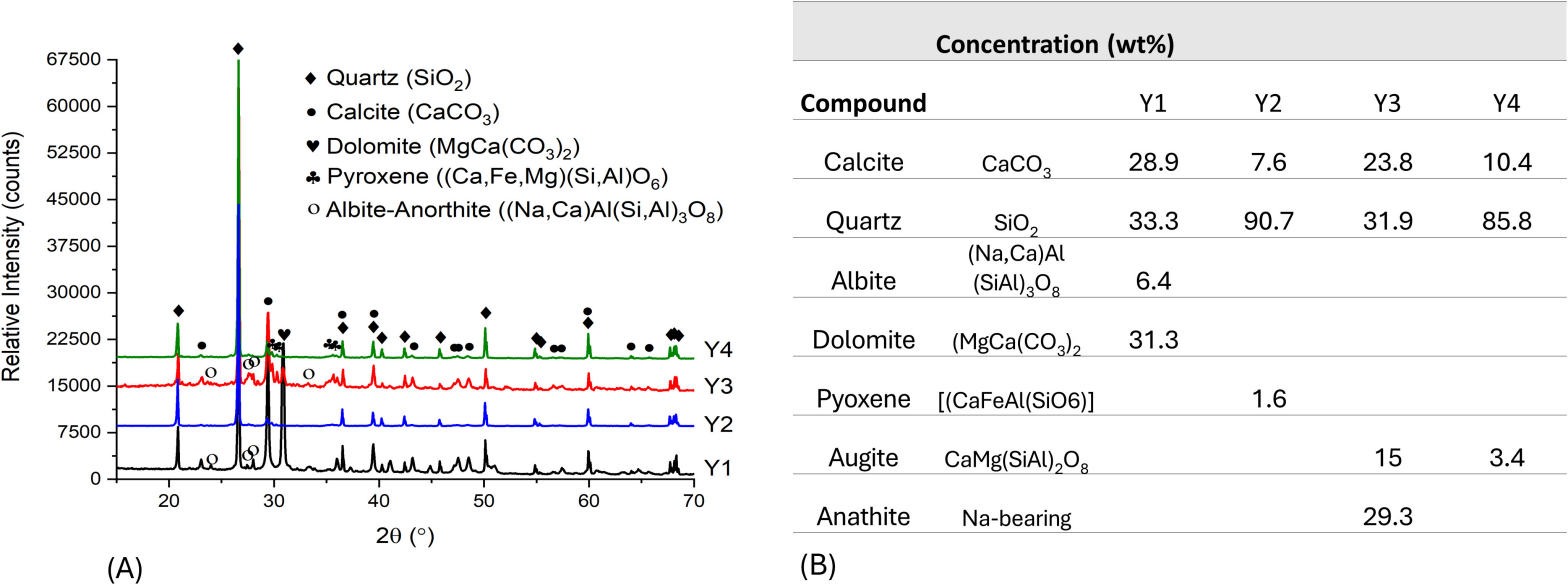
**(A)** A comparative plot of the diffractograms for the mural samples (Y1, Y2, Y3, and Y4), illustrating the XRD-detectable crystalline phases present in each sample. **(B)** Table summarizing the mineral composition.

Figure 3 illustrates the XRD analysis results of samples Y1, Y2, Y3, and Y4 that were obtained from different points of the mural materials. All the samples contain the predominant phases of quartz (SiO_2_) and calcite (CaCO_3_). Samples Y1 and Y3 have dolomite [CaMg (CO_3_)_2_], but Y4 is devoid of it. The remaining trace phases consist of silicates, specifically the pyroxene mineral augite [CaMg (SiAl)_2_O_6_] or a solid-solution mixed phase from the albite-anorthite feldspar series [(NaCa)Al(SiAl)_3_O_8_]. The present phases are consistent with the minerals of lime mortar mixed with sand. Other phases may be present that are amorphous and therefore undetectable by XRD.

#### 3.1.2 XRF analysis

Supplementary Table 4 displays the XRF analysis results, encompassing components detected at concentrations over 0.1%. Calcium and Silica are the predominant ingredients, comprising 40–50% of the mural materials. These results correspond with the knowledge of lime mortar constituents (Manoharan and Umarani, 2022).

#### 3.1.3 Raman spectroscopy analysis

**FIGURE 4:**
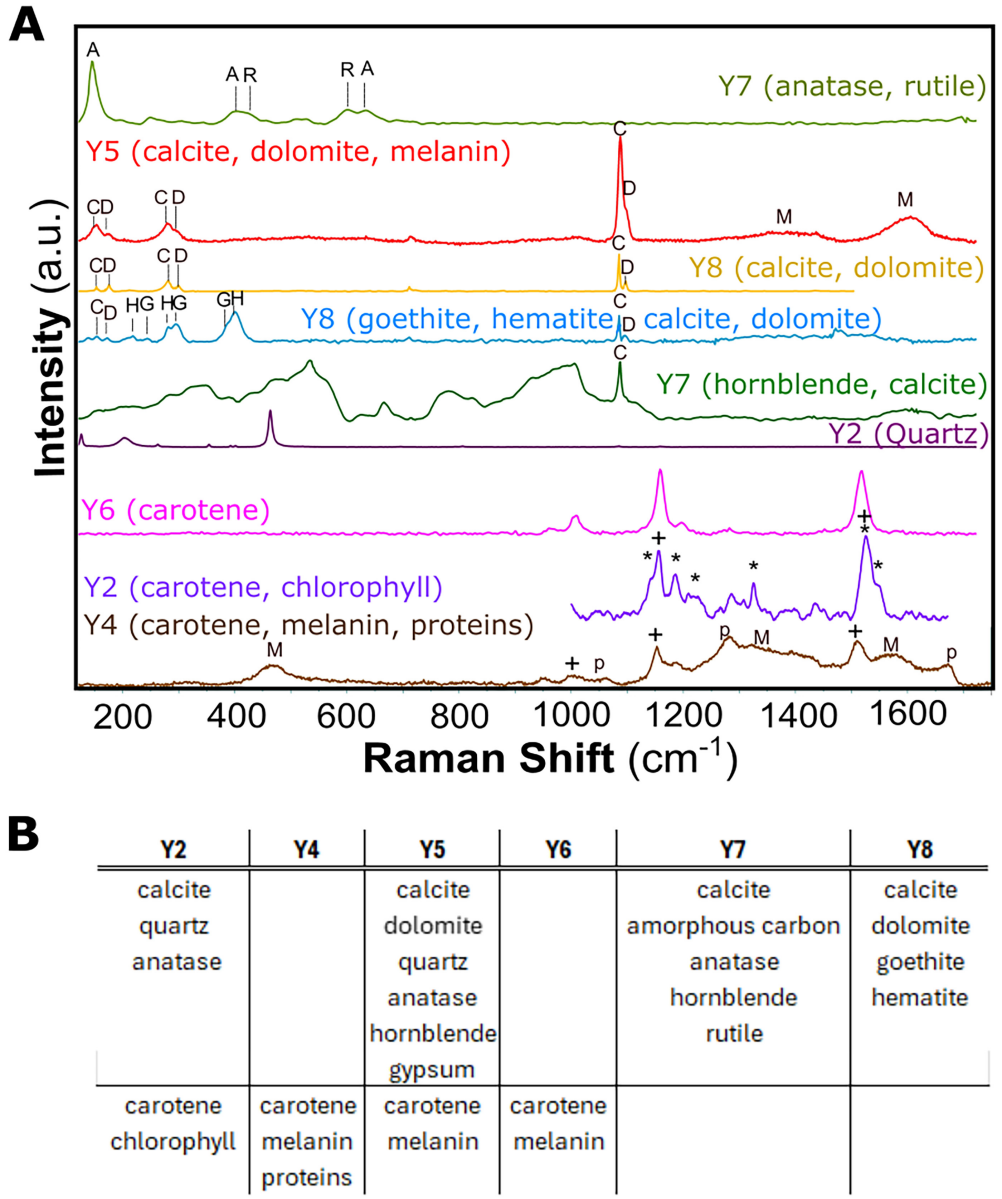
**(A)** Raman spectroscopy scattering analysis obtained from different points at Y2, Y4, Y5, Y6, Y7, and Y8 samples. The band position for every constituent is marked as follows: A, anatase; R, rutile; C, calcite; AC, amorphous carbon; G, goethite; H, hematite; D, dolomite; *, chlorophyll and +, carotene M, melanin, and p, proteins **(B)** Table of the minerals and pigments detected in each of the samples Y2, Y4, Y5, and Y8)

Figure 4 and Supplementary Table 5 present results from the Raman spectroscopy analysis of samples Y2 and Y4 that were obtained from the mural and samples Y5, Y6, Y7, and Y8 obtained from the adjacent concrete wall. The analysis identified calcite (CaCO_3_), dolomite [CaMg(CO_3_)_2_], and quartz (SiO_2_), which are the principal components of the lime mortar mixture. Additionally, the hornblende (CaNa)_2_(MgFeAl)_5_ (AlSi)_8_O_22_ (OH)_2_ silicates were determined. Other pigments and mineral phases included amorphous carbon (a main constituent in coal used as black pigment), hematite (α-Fe_2_O_3_), and goethite (α-FeOOH), typical red and yellow-brown iron oxides, as well as anatase and rutile, which are naturally occurring forms of crystalline titanium oxide (TiO_2_) commonly used in white paints. Among organic compounds, carotene, melanin, and chlorophyll were detected in samples Y2, Y4, Y5, and Y6, suggesting potential microbial colonization. In contrast, samples Y7 and Y8 showed only mineral phases, likely due to strong fluorescence interference.

### 3.2 Microscopic observations of the coating

**FIGURE 5:**
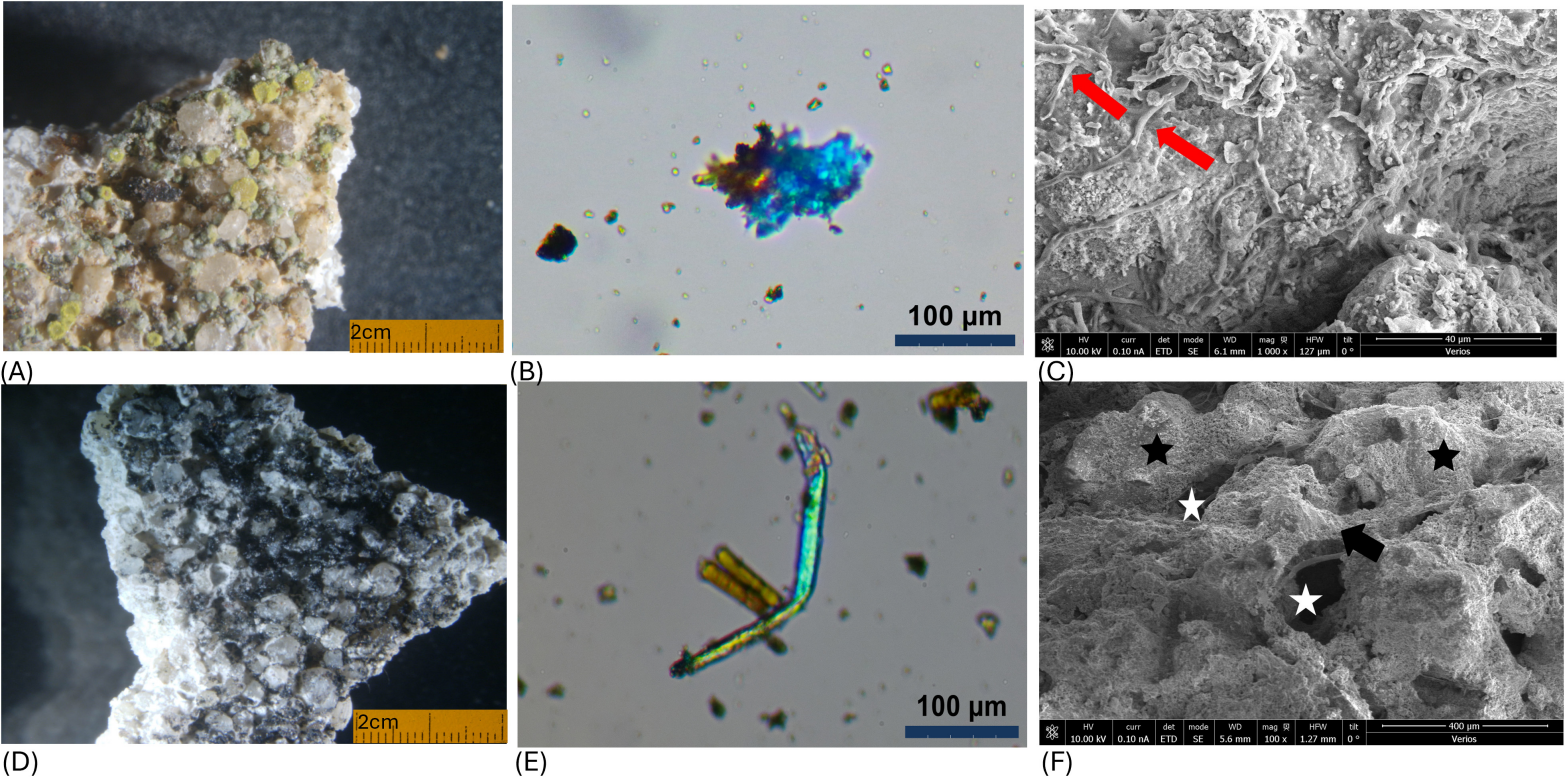
Microscopic observations of samples obtained from the wall surface: **(A,D)** Light stereomicroscope images of the biofilm with x10 magnification. **(B,E)** Images taken with cross-polarized light (XPL) demonstrate the phototrophic cells of the biofilm attached to the mortar aggregates (in light blue) and to the cement fibers. **(C)** Scanning Electron Microscope (SEM) micrographs of microbial filaments (fungi) attached to the surface of a concrete sample. **(F)** Scanning Electron Microscope image of microbial cells embedded in an amorphous matrix (black arrow) on the plaster aggregates (black asterisks). White asterisks indicate the presence of microcavities between the aggregates.

Samples of the coated building materials (e.g., mortar and cement) were examined using a range of microscopic techniques. Stereoscopic light microscope enabled observation of black round spots of fungi, lichen green-yellow thalli, and dark-green moss leaves on the surfaces of samples Y2 and Y4, respectively (Figures 5A,D). These observations indicate the presence of a well-developed microbial community where early-stage photosynthetic colonizers create conditions that allow subsequent vascular plant growth. Scanning electron microscopy provided detailed insight into the structure of the microbial coating. Figure 5C indicates filaments attached to the substrate materials. Figure 5F shows amorphous matrix binding to the substrate aggregates, which was not chemically identified in his study. Polarized light microscopy (Figures 5B,E) further distinguished organic structures (cells) from inorganic matrix components under plain and cross-polarized light, confirming microorganism attachment to the substrate materials, which in turn may cause the disaggregation of the mortar materials.

### 3.3 Characterization of the biofilm components using DNA sequencing

**FIGURE 6:**
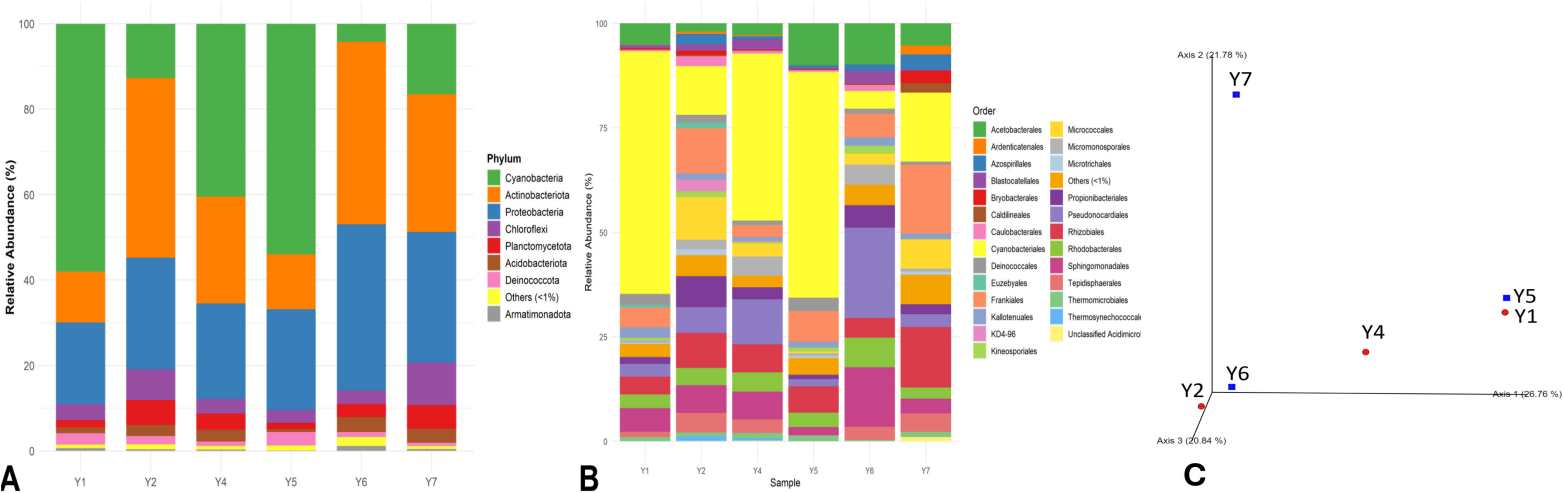
Relative abundance of bacterial communities at the phylum level **(A)** and order level **(B).** **(C)** Bray-Curtis dissimilarity PCoA plot of the bacterial communities colonizing the wall art samples (Y1, Y2, and Y4) and the exposed concrete samples (Y5, Y6, and Y7).

**FIGURE 7:**
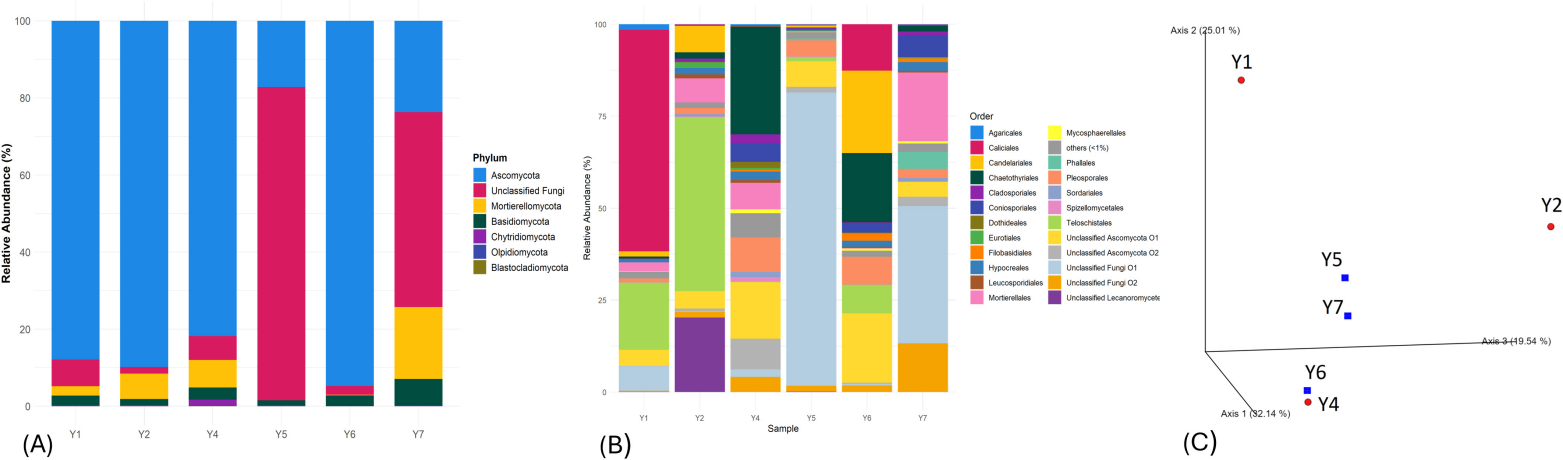
Relative abundance of fungi communities at the phylum level **(A)** and at the order level **(B)**. **(C)** Bray-Curtis dissimilarity PCoA plot of the fungi communities colonizing the wall art samples (Y1, Y2, and Y4) and the concrete samples (Y5, Y6, and Y7).

DNA was extracted from all the samples collected. Samples Y3 and Y8, however, did not produce DNA of sufficient quality for sequencing. Consequently, libraries were acquired from six samples: Y1, Y2, Y4 (mural), and Y5, Y6, and Y7 (concrete). A total of 182,545 high-quality sequences were obtained from the 16S rRNA gene libraries after quality filtering, denoising, and chimera removal, with read counts ranging from 20,824 to 48,251 per sample (mean = 30,424). For the ITS region, a total of 99,724 high-quality sequences were retained after processing, with read counts ranging from 13,748 to 23,317 per sample (mean = 16,621). Statistical analysis revealed that each sample contained a variety of microbes, and there were no specific groupings based on the types of materials used (e.g., lime mortar and concrete) (Figures 6C, 7C).

#### 3.3.1 Bacterial taxa diversity

The study revealed 20 bacterial phyla and 181 bacterial genera in six of the eight samples: Y1, Y2, Y4, Y5, Y6, and Y7. The analysis of the bacterial communities found four main groups: Cyanobacteria, Actinobacteria, Proteobacteria, and Chloroflexi, which together made up about 88–92% of all the bacteria, along with a few other groups that each made up less than 5% of the total. About 1–2% of the ASVs obtained were not assigned to any specific taxa and are marked as “others” (Figure 6). The Phylum Cyanobacteria dominates samples Y1, Y4, and Y5, accounting for 40–58%, while Actinobacteria dominates samples, Y2, Y6, and Y7, accounting for 35–45%. The phylum Proteobacteria made up about 20–38% of the total, and the Chloroflexi phylum accounted for 3–10% of the total bacterial types (Figure 6A). All the samples revealed Cyanobacteriales as the dominant order within the Cyanobacteria (Figure 6B). The family-level identification revealed two main groups that were found in all the samples. Among these groups, the Chroococcidiopsaceae family was found in amounts ranging from 8% in sample Y4 to 45% in sample Y2, while the Nostocaceae family was found in smaller amounts, from 1% in sample Y7 to 30% in sample Y6. In the Actinobacteria group, five families were the most common: Geodermatophilaceae, Micromonosporaceae, Nocardioidaceae, Pseudonocardiaceae, and Micrococcaceae, which constitute about 72–84% of the Actinobacteria community. The dominant class in samples Y1, Y4, and Y7 was Geodermatophilaceae (35–48%), while in samples Y6 and Y4, the Pseudonocardiaceae class was the most dominant (40–50%) (Figure 6B and Supplementary Table 6).

#### 3.3.2 Fungal taxa diversity

About 2–50% of the total ASVs retrieved were unidentified. All the samples included the Ascomycota phylum among the identified taxa. In samples Y1, Y2, Y4, and Y6, the Ascomycota phylum was dominant and present in more than 80% of the fungal taxa. (In samples Y1 and Y6, it is present in more than 95% of the taxa). All samples exhibited a relatively low abundance of the phylum Mortierellomycota, ranging from 2 to 18%. Approximately 1–7% of all the samples contained representatives from the Basidiomycota. The phyla Chytridiomycota, Olpidiomycota, and Blastocladiomycota were present in less than 1% of all the samples (Figure 7A). At the order level, ASVs’ distribution varied among sites. All the samples tested showed lichenized fungi in the Lecanoromycetes group (Ascomycota), especially in samples Y1 (90%) and Y2 (80%). The distribution of the Candelariomycetes, Eurotiomycetes, and Dothideomycetes was detected mostly in the samples Y4, Y6, and Y7 (Figure 7B and Supplementary Table 7).

#### 3.3.3 Comparison between microbial colonization on the different substrates

Principal Coordinate Analysis (PCoA) was performed on the six samples to explore differences in microbial community composition. Originally, eight samples were collected, but two failed to yield DNA of sufficient quality for sequencing, resulting in a reduced sample size. The six samples present two groups regarding the substrate material, mortar or cement. The analysis revealed no clear separation between the samples in the ordination space (Figures 6C, 7C). Additionally, PERMANOVA analysis using Bray–Curtis dissimilarity indicated no significant compositional differences between substrate types for either bacterial (*p* = 0.518) or fungal (*p* = 0.743) communities (Supplementary Table 8). Consistently, Kruskal–Wallis tests of alpha diversity showed no significant differences between cement and rock samples for observed features (bacteria: *H* = 0.048, *p* = 0.827; fungi: *H* = 1.19, *p* = 0.275) or Shannon entropy (bacteria: *H* = 0.048, *p* = 0.827; fungi: *H* = 0.43, *p* = 0.513). Although individual samples varied in richness and diversity, no substrate-specific patterns were detected. Together, these results indicate that microbial diversity is not structured by substrate type and is more likely influenced by local microenvironmental heterogeneity.

## 4 Discussion

Since 1971, the “Come, Peace” sgraffito has undergone progressive weathering. Close examination showed a mature microbial layer incorporating lichens and mosses, reaching ∼0.5 cm thickness in several areas consistent with conditions favoring biofilm development (e.g., humidity, substrate porosity) (Liu et al., 2022; Sanmartín et al., 2021b). Moreover, the SEM study pointed to microbial cells, filaments, and hyphae adhering to construction particles. This may result in the removal of material grains from the substrate, thereby weakening its integrity over time. The chemical tests on the samples from the damaged sgraffito showed that they mainly contained two substances: silica oxide (quartz) and calcium carbonate (calcite). Silica oxide and calcium carbonate are known as the major components comprising lime mortars and cement concrete. Interestingly, the analytical examination’s findings point to elevated concentrations of iron oxides in the art materials. This finding may be explained by the assumption that the sand used for the lime mortar mixture originated from Nubian sandstone sourced from the Negev Desert (Farkash and Tal, 2017; Weissbrod and Sneh, 1997).

Among the bacterial domain, Cyanobacteria, Actinobacteria, and Proteobacteria were detected as the principal bacterial taxa. This discovery agrees with established research concerning the bacterial communities surviving in building materials (Dyda et al., 2019; Kukletová and Chromková, 2018). The Cyanobacteria group is crucial in biofilm formation as the primary energy producer in this nutrient-deficient environment. Interestingly, *Literella*, a cyanobacterium genus that was recently described within the fissures and cracks of granite stones and rocks were detected in all the samples (Jung et al., 2020). Four of the samples contained members of the filamentous cyanobacterium genus *Scytonema* which is already known as a colonizer of black crusts on building materials (De Miguel et al., 1995; Gaylarde et al., 2004; Gaylarde et al., 2007). *Nostoc* is a genus of filamentous cyanobacteria that can survive on stone monuments due to their ability to remain desiccated for months or years (Ramírez et al., 2011). All the samples collected included members of the Actinobacteria group, such as *Blastococcus*, *Nocardioides*, *Micromonosporaceae*, and *Pseudonocardia*. Furthermore, we identified representatives from *Modestobacter*, *Micrococcaceae*, and *Geodermatophilus* in five of the samples. Actinobacteria, a prevalent soil bacterium, are recognized as a significant group residing in building materials (Abdulla et al., 2008; Saadouli et al., 2022; Urzì et al., 2004).

Concerning the fungi, the most represented phylum on the examined walls is Ascomycota. Within the Ascomycota phylum, the most prevalent groups were Lecanoromycetes, Dothideomycetes, and Euromycetes. Lecanoromycetes are lichen-forming fungi with a worldwide range, predominantly found in cryptoendolithic colonies that inhabit stones and rocks in both natural environments and man-made structures (Cozzolino et al., 2021). In our study, we found the *Teloschistaceae*, a substantial family of lichen-forming fungi, colonizing both the lime mortar and concrete samples. Their ability to cause damage to building materials has been recorded in marble structures, where they may infiltrate up to 10 mm into the stone and disintegrate the marble surface (Fuentes et al., 2021). The results revealed other important taxa of Ascomycetes: Dothideomycetes and Eurotiomycetes, which are part of the black meristematic fungal communities (MCF) (De Leo et al., 2019; Selbmann et al., 2015; Sert et al., 2007). Members of this fungal group, such as *Knufia, Coniosporium*, and *Cladosporium*, were detected in all samples and are already known for their ability to cause damage by penetrating, degrading, and discoloring the stone surface. The production of protective pigments, such as melanin and carotenoids, revealed in various samples (Figure 4), further enhances their resilience (Erdmann et al., 2022; Isola et al., 2015). Most rock-inhabiting fungi can infiltrate between crystal structures (Figure 5E; Knabe and Gorbushina, 2018; Rabbachin et al., 2024). Interestingly, we identified taxa commonly referred to as sooty-mold fungi, such as representatives from the Trichomeriaceae and Capnodiaceae families (Chomnunti et al., 2014). Their presence may indicate an alternative pathway for biofilm formation on the wall decoration materials (Harding et al., 2009). We link this phenomenon to a large tree that covered much of the wall surface before conservation work began. The tree harbors a species of leaf aphids that feed on its sap and secrete a sugary substance known as honeydew (Meng et al., 2023).

Despite the disparity in material composition, which might have resulted in variations in microbial populations, no significant difference was found between the groups. This may be because over the years, the biofilm was well developed, containing microorganisms from all domains. Another reason could be attributed to the differentiation of microclimatic conditions along the wall. In regions shaded by the adjacent Pistacia tree (*Pistacia palaestina*), the coating appeared dark to black, while in locations with slightly greater sun exposure, it also exhibited green patches (Figure 2). Interestingly, a recent study of epilithic biofilms on Stone Dog sculptures on the Leizhou Peninsula, China, found that despite identical exposure within a small courtyard, the biofilms exhibited high biodiversity and distinct community compositions (Sanmartín et al., 2021a). This underscores the critical influence of microenvironmental conditions and the need for precise microclimatic measurements, including radiation and humidity (Del Mondo et al., 2021; Sanmartín et al., 2021a).

## 5 Conclusion

The sgraffito wall decoration has experienced substantial deterioration and weathering since its creation, significantly diminishing the visibility of the artwork. Analytical results revealed a well-established, diverse microbial community, including fungi, bacteria, lichens, and mosses attached to the substrate. This process may reduce the structural integrity of the wall-art materials. Notably, variations in microbial composition appear to be more closely linked to localized microclimatic conditions than to the substrate materials themselves. Thus, future studies should address microclimatic measures, physicochemical characteristics, and metabolic functions of the microbial communities. Due to its climatic exposure and porous composition, the artwork remains vulnerable to microbial re-colonization. Therefore, sustained biological monitoring combined with conservation expertise and community engagement is essential for its long-term preservation.

## Data Availability

The data presented in this study are publicly available. The data can be found here: https://www.ncbi.nlm.nih.gov/bioproject/PRJNA1265466/, accession number PRJNA1265466.

## Footnotes

1
https://ims.gov.il/en/node/310
