# Correction: Extensive biofilm covering on sgraffito wall art: a call for proactive monitoring

**DOI:** 10.3389/fmicb.2026.1795378

**Published:** 2026-02-09

**Authors:** Irit Nir, Anath Sharaby, Hana Barak, Mariela J. Pavan, Lonia R. Friedlander, Victor Multanen, Ariel Kushmaro

**Affiliations:** 1 Avram and Stella Goldstein-Goren Department of Biotechnology Engineering, Ben-Gurion University of the Negev, Beer Sheva, Israel; 2 Ilse Katz Institute for Nanoscale Science and Technology, Ben- Gurion University of the Negev, Beer Sheva, Israel; 3 The Goldman Sonnenfeldt School of Sustainability and Climate Change, Ben-Gurion University of the Negev, Beer Sheva, Israel

**Keywords:** sgraffito, wall art, lime mortar, bio-weathering, next-generation sequencing (NGS)

**Error in Figure 2**

There was a mistake in Figure 2 as published. The sample numbers/location Y1, Y2, Y3, and Y4 were missing from the upper section of Figure 2. The corrected Figure 2, with the sample numbers Y1, Y2, Y3, and Y4, appears below.

**Figure 2:**
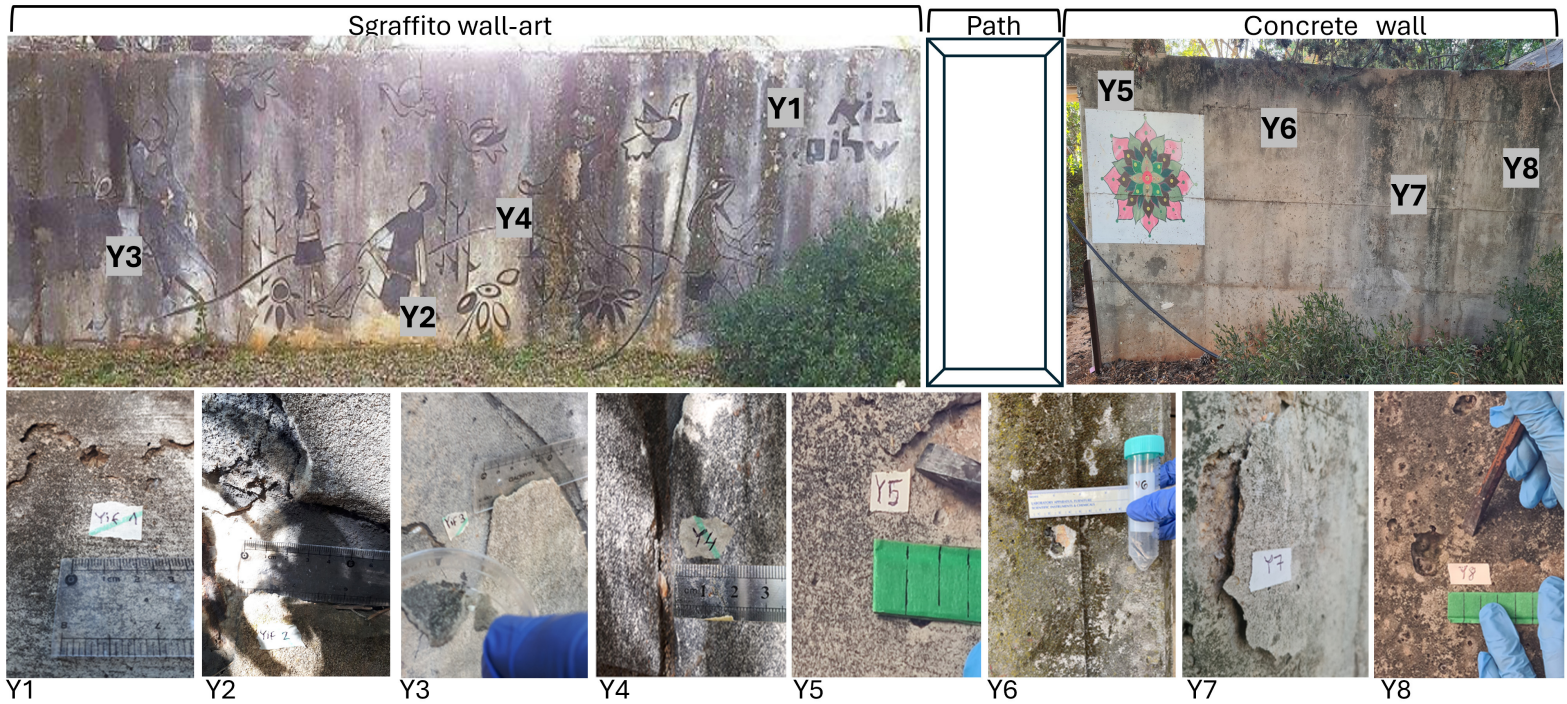
Sampling site: the “Sgraffito” wall art and the adjacent concrete wall, indicating the positions of sampling points Y1-Y8 (above), together with close-up photographs of the samples collected in this study (at the lower portion of the figure).

The original version of this article has been updated.

