# Supplementary material for: Extensive biofilm covering on sgraffito wall art: a call for proactive monitoring
Source: Front Microbiol. 2026 Jan 21;16:1664404. doi: 10.3389/fmicb.2025.1664404 (PMC12869997; doi:10.3389/fmicb.2025.1664404)
Supplement: Supplementary file 1 [file Supplementary_file_1.pdf]

Table S1: Temperature and precipitation monitoring at Kibbutz Yiftach meteorological station (Israel) (33.1259°N, 35.5521°E). Acquired from the Israel Meteorological Service (IMS) website (https://ims.gov.il/en, accessed 15.11.2025)

Table S1.1: Monthly Average Temperature (C^0) monitoring from 2015 to 2024

**Month/Year**	**2015**	**2016**	**2017**	**2018**	**2019**	**2020**	**2021**	**2022**	**2023**	**2024**
**1**	10.7	10.5	10.7	11.2	11.1	10.1	14.2	12.1	13.8	14.5
**2**	12.2	15.4	12.1	15	12.2	12.1	13.3	13.1	11.7	13.5
**3**	16.2	16.5	15.1	17.7	13	15.1	14.3	12.3	15.7	16.9
**4**	17.5	21.5	19	19.8	16.4	17	19.8	17.9	17.1	19.5
**5**	20.8	22.4	22.5	24.6	24.4	23.4	20.4	20.1	20	21.1
**6**	23.7	27	25.1	25.6	26.1	24	22	22.9	23	25
**7**	26.7	28.5	28.8	27.1	26.7	27.2	26.4	25.2	25.5	27.1
**8**	28.9	27.8	27.4	27.1	27.2	27.6	26.8	26	27.7	27
**9**	28.5	25.6	26.5	26.3	25.9	29.7	24.8	25.3	24.9	23.6
**10**	24.2	24.4	22.7	23.4	24.3	26.4	21.9	22.7	23.5	21.4
**11**	18.6	19	18.5	18.5	20.8	18.3	18.7	18.7	19.2	17.7
**12**	12.9	10.8	15.9	12.7	18.9	15	15.6	14.3	16.2	13.8
**Average/year**	**20.08**	**20.78**	**20.36**	**20.75**	**20.58**	**20.49**	**19.97**	**19.04**	**19.96**	**20.41**

Table S1.2: Monthly and Yearly Average Rainfall (mm) Monitoring from 2000 to 2024

Month	Rain (mm)	Year	Rain (mm)
01/2000	278.5	2000	548
02/2000	62	2001	391
03/2000	54	2002	478
04/2000	9.3	2003	766
05/2000	0	2004	598.4
09/2000	5	2005	527.4
10/2000	31.2	2006	482.2
11/2000	44.2	2007	456
12/2000	100.1	2008	364.3
01/2001	69.6	2009	614.1
02/2001	100.7	2010	370.9
03/2001	15.3	2011	617.5
04/2001	2.6	2012	770.2
05/2001	1.8	2013	461.8
09/2001	0	2014	363.3
10/2001	22.2	2015	596.5
11/2001	47.2	2016	505.1
12/2001	132	2017	242.3
01/2002	152	2018	647.6
02/2002	79	2019	802.1
03/2002	109.7	2020	685.9
04/2002	48.2	2021	471
05/2002	1.3	2022	512.2
09/2002	0	2023	520.1
10/2002	3.8	2024	575.6
11/2002	53.1	Average	**534.7**
12/2002	183.8		
01/2003	137		
02/2003	296.5		
03/2003	141.6		
04/2003	21.2		
05/2003	0.8		
10/2003	3		
11/2003	21.9		
12/2003	144		
01/2004	271.2		
02/2004	128.2		
03/2004	9.3		
04/2004	12.9		

05/2004	0.3		
09/2004	0		
10/2004	0.7		
11/2004	134.8		
12/2004	41		
01/2005	127.3		
02/2005	141.4		
03/2005	17.5		
04/2005	4.8		
05/2005	24.3		
08/2005	0		
09/2005	0		
10/2005	24.8		
11/2005	69.9		
12/2005	117.4		
01/2006	98.6		
02/2006	79.5		
03/2006	13.1		
04/2006	114		
05/2006	0.1		
08/2006	21.1		
09/2006	0		
10/2006	95.8		
11/2006	20.5		
12/2006	39.5		
01/2007	115.1		
02/2007	133.8		
03/2007	56.9		
04/2007	21.8		
05/2007	6.9		
09/2007	0		
10/2007	0.2		
11/2007	60.6		
12/2007	60.7		
01/2008	124.3		
02/2008	67.1		
03/2008	14.7		
04/2008	0.1		
05/2008	5.6		
08/2008	3.7		

09/2008	0.2		
10/2008	24.6		
11/2008	57.9		
12/2008	66.1		
01/2009	58.7		
02/2009	173.2		
03/2009	66.4		
04/2009	22.7		
05/2009	0.4		
09/2009	21.4		
10/2009	38.2		
11/2009	112		
12/2009	121.1		
01/2010	135.2		
02/2010	75.6		
03/2010	1.4		
04/2010	0.5		
05/2010	0.8		
09/2010	0		
10/2010	8.2		
11/2010	0		
12/2010	149.2		
01/2011	88.1		
02/2011	176.1		
03/2011	88.8		
04/2011	76.1		
05/2011	22.2		
06/2011	0.3		
09/2011	64		
10/2011	2.8		
11/2011	62.8		
12/2011	36.3		
01/2012	230.6		
02/2012	159.2		
03/2012	77		
04/2012	0		
05/2012	5.6		
09/2012	0		
10/2012	12.7		
11/2012	81.5		

12/2012	203.6		
01/2013	207.2		
02/2013	47.4		
03/2013	13.6		
04/2013	46.9		
05/2013	1.3		
08/2013	0		
09/2013	0.3		
10/2013	6.1		
11/2013	8.9		
12/2013	130.1		
01/2014	22.5		
02/2014	35.1		
03/2014	75.3		
04/2014	1.5		
05/2014	52.5		
06/2014	0		
08/2014	0		
09/2014	2		
10/2014	14.2		
11/2014	127.2		
12/2014	33		
01/2015	172.1		
02/2015	140.9		
03/2015	29.5		
04/2015	56.8		
05/2015	1.1		
06/2015	0		
08/2015	0		
09/2015	0.3		
10/2015	82.4		
11/2015	53.4		
12/2015	60		
01/2016	118		
02/2016	74.9		
03/2016	33.7		
04/2016	27		
05/2016	3		
08/2016	0		
09/2016	0.1		

10/2016	4.1		
11/2016	7.9		
12/2016	236.4		
01/2017	99.4		
02/2017	16.6		
03/2017	43.6		
04/2017	4.9		
05/2017	1.6		
08/2017	0		
09/2017	0		
10/2017	12.7		
11/2017	16.4		
12/2017	47.1		
01/2018	242.8		
02/2018	81.5		
03/2018	7.7		
04/2018	16.7		
05/2018	19.6		
06/2018	2.5		
08/2018	0		
09/2018	0		
10/2018	89.8		
11/2018	28.1		
12/2018	158.9		
01/2019	202.8		
02/2019	174.5		
03/2019	116.6		
04/2019	39.3		
05/2019	0		
06/2019	0.1		
08/2019	0		
09/2019	0		
10/2019	53.3		
11/2019	5.4		
12/2019	210.1		
01/2020	267.2		
02/2020	95.1		
03/2020	67.4		
04/2020	28.4		
05/2020	30.8		

08/2020	0		
09/2020	0		
10/2020	0		
11/2020	138.4		
12/2020	58.6		
01/2021	182.7		
02/2021	75.2		
03/2021	34.2		
04/2021	17.5		
05/2021	0		
08/2021	0		
09/2021	0.5		
10/2021	10.3		
11/2021	22.4		
12/2021	128.2		
01/2022	192.2		
02/2022	99.6		
03/2022	115.1		
04/2022	0		
05/2022	12.9		
06/2022	0		
08/2022	0		
09/2022	0		
10/2022	4.4		
11/2022	46		
12/2022	42		
01/2023	68.3		
02/2023	139.8		
03/2023	53.7		
04/2023	50.3		
05/2023	0.5		
06/2023	0.8		
08/2023	0		
09/2023	0		
10/2023	19.2		
11/2023	79.2		
12/2023	108.3		
01/2024	226.5		
02/2024	85.1		
03/2024	46		

04/2024	56.7		
05/2024	4.9		
08/2024	0		
09/2024	0.2		
10/2024	0		
11/2024	58		
12/2024	98.2		
