# Supplementary material for: Extensive biofilm covering on sgraffito wall art: a call for proactive monitoring
Source: Front Microbiol. 2026 Jan 21;16:1664404. doi: 10.3389/fmicb.2025.1664404 (PMC12869997; doi:10.3389/fmicb.2025.1664404)
Supplement: Supplementary file 2 [file Supplementary_file_2.pdf]

Table S2: Description of the experimental methods related to the samples obtained

Sample	Section	Stereoscopic microscope images	Sufficient quality of DNA & sequencing	XRD/ XRF	Raman Spectroscopy	SEM
Y1	Wall art	+	+	+	-	+
Y2	Wall art	+	+	+	+	+
Y3	Wall art	+	-	+	-	+
Y4	Wall art	+	+	+	+	+
Y5	Concrete	+	-		+	+
Y6	Concrete	+	+		+	+
Y7	Concrete	+	+		+	+
Y8	Concrete	+	+		+	+
