# Supplementary figures and images for: Extensive biofilm covering on sgraffito wall art: a call for proactive monitoring
Source: Front Microbiol. 2026 Jan 21;16:1664404. doi: 10.3389/fmicb.2025.1664404 (PMC12869997; doi:10.3389/fmicb.2025.1664404)

S3: Stereomicroscopic Images of samples Y1-Y8

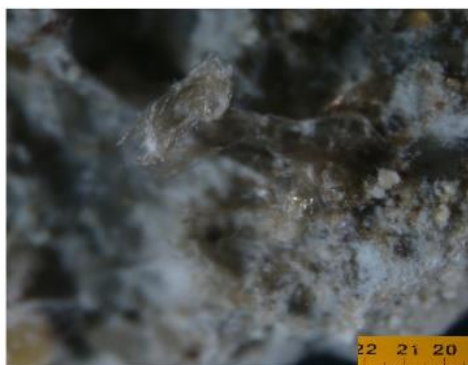

Y1

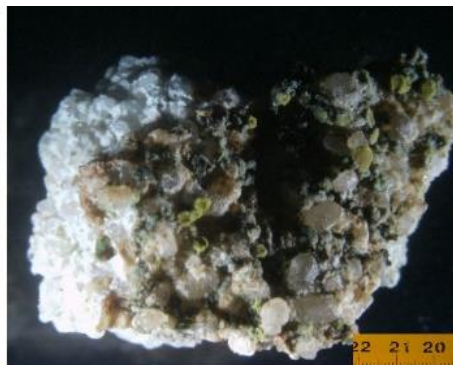

Y2

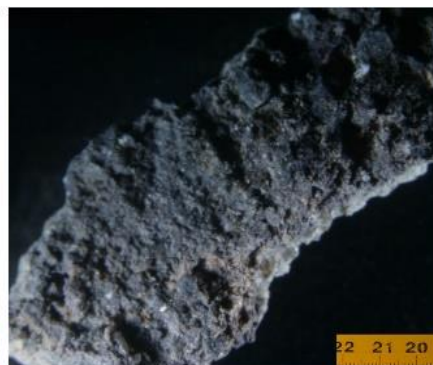

Y3

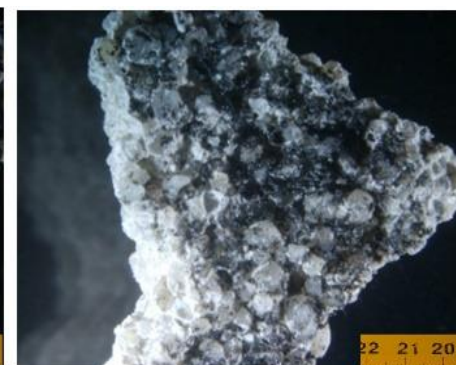

Y4

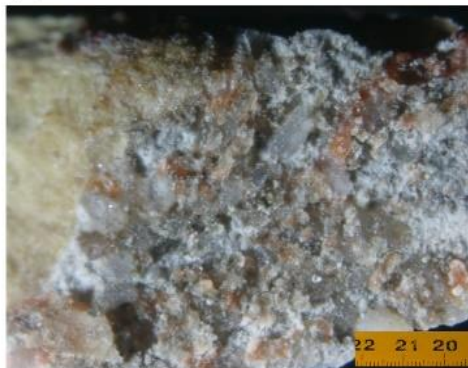

Y5

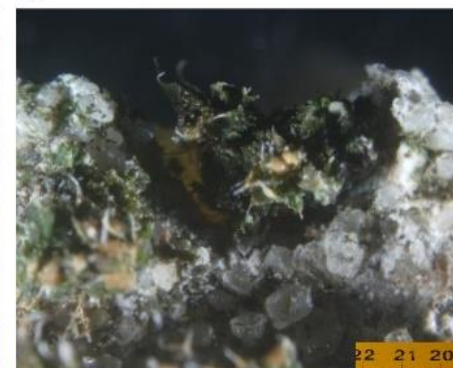

Y6

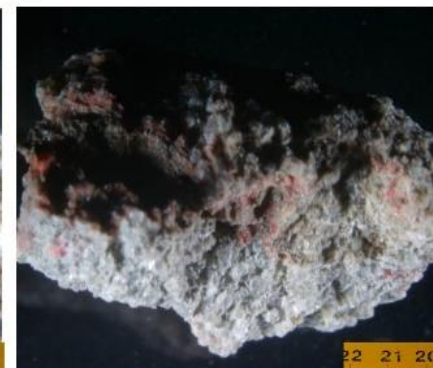

Y7

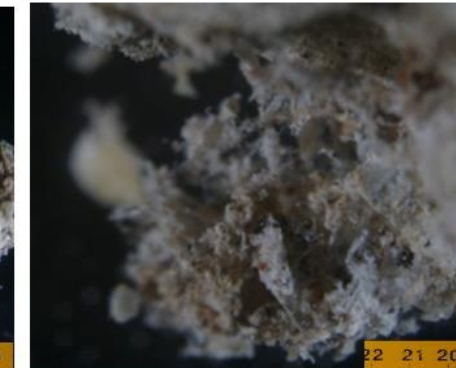

Y8

Supplement: Supplementary file 3 [file Supplementary_file_3.pdf]
