# Supplementary material for: Extensive biofilm covering on sgraffito wall art: a call for proactive monitoring
Source: Front Microbiol. 2026 Jan 21;16:1664404. doi: 10.3389/fmicb.2025.1664404 (PMC12869997; doi:10.3389/fmicb.2025.1664404)
Supplement: Supplementary file 4 [file Supplementary_file_4.pdf]

Table S4: XRF analysis results

Table S4.1: Element concentrations (> 0.2%) in samples Y1-Y4 revealed by XRF analysis

Sample	Y1	Y2	Y3	Y4
Ca	59.019	14.432	35.171	20.051
Si	26.964	71.638	35.457	63.558
Al	5.609	1.525	8.767	4.789
Mg	4.169	---	3.481	1.465
Fe	2.081	10.137	8.437	5.222
K	0.716	0.360	1.366	0,545
Na	0.308	---	3.677	2.081
Ti	0.325	1.000	1.426	0.902

Table S4.2: Raw data of XRF analysis samples Y1-Y4

9/16/2025 2:11:14 PM<br>PANalytical<br>Quantification of sample 888u_**Y2**<br>Normalised to: 100.0 %<br>Sample type: Loose powder<br>Correction applied for medium: Yes<br>Correction applied for film: Yes<br>Element Conc (%).<br>1 Si 71.638<br>2 Ca 14.432<br>3 Fe 10.137<br>4 Al 1.525<br>5 Ti 1.000<br>6 S 0.408<br>7 K 0.360<br>8 Sr 0.203<br>9 Mn 0.158<br>10 Zr 0.092<br>11 Zn 0.048	5/8/2023 2:17:38 PM<br>PANalytical<br>Quantification of sample 234u_Y-1<br>Normalised to: 100.0 %<br>Sample type: Loose powder<br>Correction applied for medium: Yes<br>Correction applied for film: Yes<br>Element Conc (%).<br>1 Ca 59.019<br>2 Si 26.964<br>3 Al 5.609<br>4 Mg 4.169<br>5 Fe 2.081<br>6 K 0.716<br>7 S 0.511<br>8 Ti 0.325<br>9 Na 0.308<br>10 Sr 0.084<br>11 Cl 0.078<br>12 P 0.058<br>13 Mn 0.041<br>14 Cr 0.022<br>15 Zr 0.013

5/8/2023 2:22:09 PM<br>PANalytical<br>Quantification of sample 234u_**Y-3**<br>Normalised to: 100.0 %<br>Sample type: Loose powder<br>Correction applied for medium: Yes<br>Correction applied for film: Yes<br>Element Conc.(%)<br>1 Si 35.457<br>2 Ca 35.171<br>3 Al 8.767<br>4 Fe 8.437<br>5 Mg 3.841<br>6 Na 3.677<br>7 Ti 1.426<br>8 K 1.366<br>9 S 0.698<br>10 P 0.540<br>11 Cl 0.299<br>12 Mn 0.133<br>13 Sr 0.061<br>14 V 0.042<br>15 Cr 0.038<br>16 Pb 0.022<br>17 Zn 0.01	5/8/2023 2:52:13 PM<br>PANalytical<br>Quantification of sample 234u_**Y-4**<br>Normalised to: 100.0 %<br>Sample type: Loose powder<br>Correction applied for medium: Yes<br>Correction applied for film: Yes<br>Element Conc.(%)<br>1 Si 63.558<br>2 Ca 20.051<br>3 Fe 5.222<br>4 Al 4.789<br>5 Na 2.081<br>6 Mg 1.465<br>7 Ti 0.902<br>8 K 0.545<br>9 P 0.511<br>10 S 0.463<br>11 Cl 0.109<br>12 Zr 0.091<br>13 Mn 0.088<br>14 Sr 0.060<br>15 Cr 0.038<br>16 V 0.015<br>17 Zn 0.013
