# Supplementary material for: Extensive biofilm covering on sgraffito wall art: a call for proactive monitoring
Source: Front Microbiol. 2026 Jan 21;16:1664404. doi: 10.3389/fmicb.2025.1664404 (PMC12869997; doi:10.3389/fmicb.2025.1664404)
Supplement: Supplementary file 5 [file Supplementary_file_5.pdf]

Table S5: Micrographs and additional Raman spectra

Raman spectra obtained from different samples are shown below (black or blue spectra). The corresponding fitting using the KnowItAll database is shown in orange and green.

Sample	Micrographs	Raman spectra
**Y2**	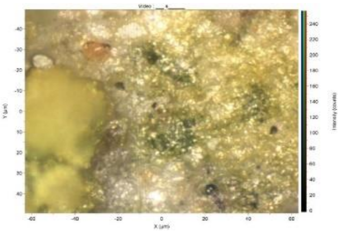 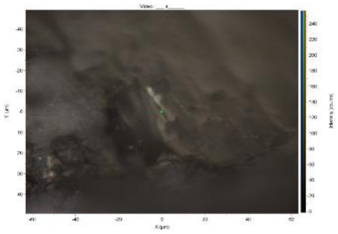	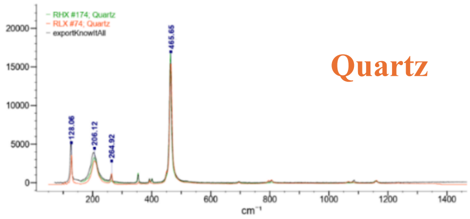 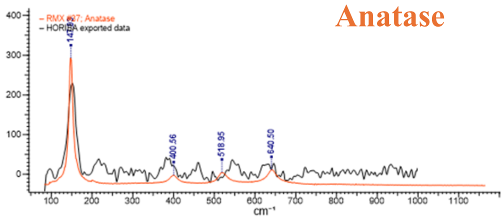 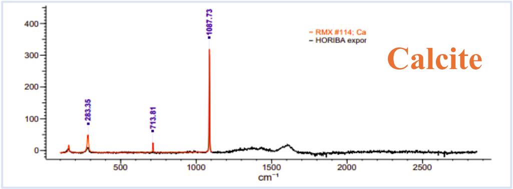
**Y4**	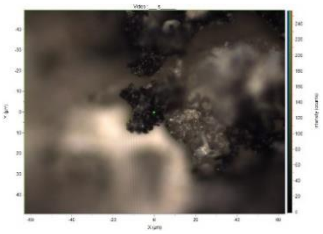	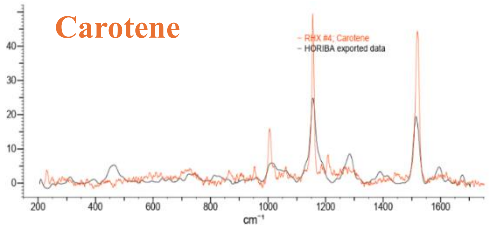

<table>
<tr>
<td>Y5</td>
<td>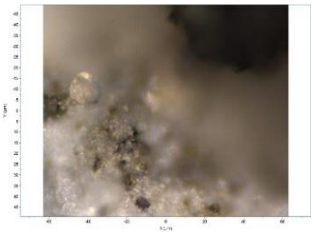</td>
<td>
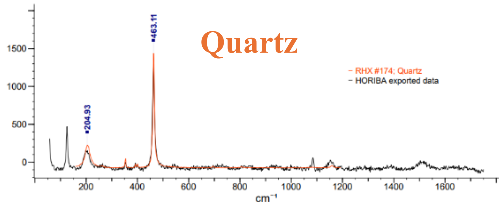

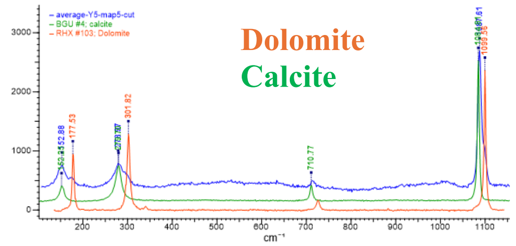

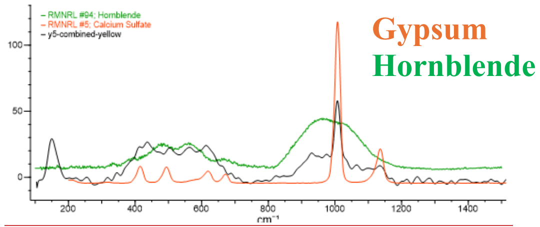

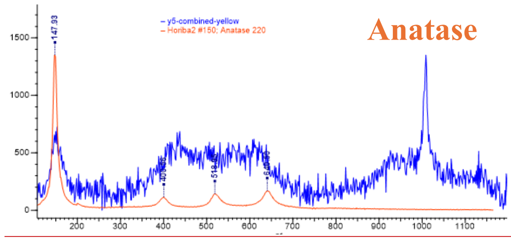

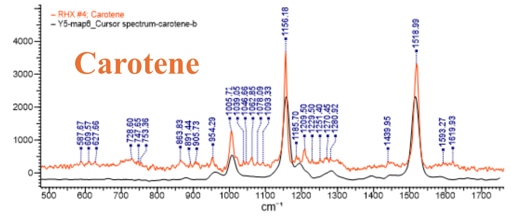

</td>
</tr>
<tr>
<td>Y6</td>
<td>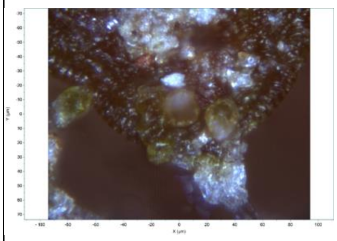</td>
<td>
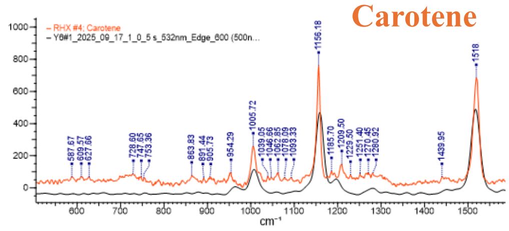

</td>
</tr>
</table>

**Y7**	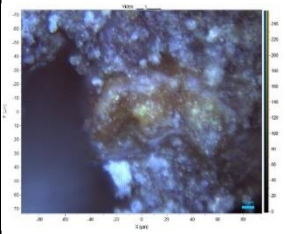	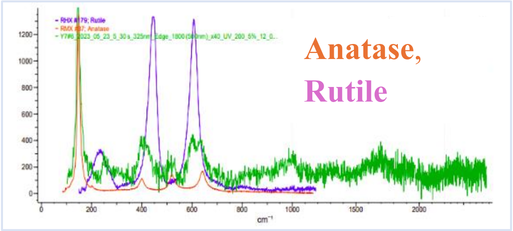<br>(The Raman spectrum obtained from Y7 is shown in green).
**Y8**	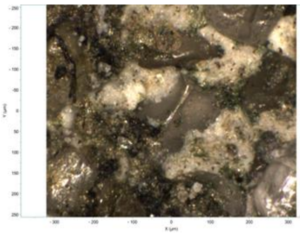	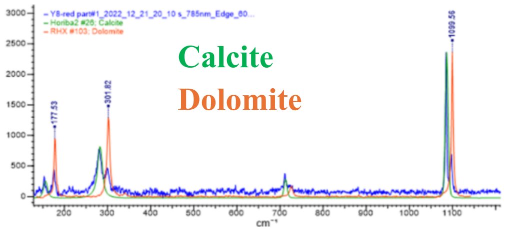
