# Supplementary material for: Extensive biofilm covering on sgraffito wall art: a call for proactive monitoring
Source: Front Microbiol. 2026 Jan 21;16:1664404. doi: 10.3389/fmicb.2025.1664404 (PMC12869997; doi:10.3389/fmicb.2025.1664404)
Supplement: Supplementary file 8 [file Supplementary_file_8.pdf]

Table S8: Statistical analysis

S8.A. Bacterial community analysis

Table S8.1. Bray-Curtis dissimilarity PERMANOVA results for bacterial community (16S rRNA gene) analysis.

Sample size	6
Number of groups	2
Test statistics (pseudo-F)	0.917
p-value	0.518
Permutations number	999

Table S8.2. Bray-Curtis dissimilarity matrix for bacterial communities (16S rRNA gene)

	Y1	Y2	Y4	Y5	Y6	Y7
Y1	0	0.94	0.8688	0.6982	0.9467	0.9484
Y2	0.94	0	0.9296	0.9557	0.9345	0.9514
Y4	0.8688	0.9296	0	0.889	0.9488	0.9514
Y5	0.6982	0.9557	0.889	0	0.9402	0.9417
Y6	0.9467	0.9345	0.9488	0.9402	0	0.9581
Y7	0.9484	0.9514	0.9514	0.9417	0.9581	0

Table S8.3. Alpha diversity indices for bacterial communities. Observed features and Shannon entropy per sample, with Kruskal-Wallis test results (Cement Y5 Y6 Y7 (n=3), Rock Y1 Y2 Y4 (n=3)).

	Observed_features	Shannon_entropy
Y1	1210	9.67
Y2	599	8.42
Y4	1515	9.81
Y5	1867	10.29
Y6	751	8.53
Y7	949	9.00
H	0.048	0.048
P-value	0.827	0.827

S8.B. Fungal community analysis

Table S8.4. Bray-Curtis dissimilarity PERMANOVA results for fungal community (ITS gene) analysis.

Sample size	6
Number of groups	2
Test statistics (pseudo-F)	0.833
p-value	0.743
Permutations number	999

Table S8.5. Alpha diversity indices for fungal communities. Observed features and Shannon entropy per sample, with Kruskal-Wallis test results (Cement Y5 Y6 Y7 (n=3), Rock Y1 Y2 Y4 (n=3)).

	Y1	Y2	Y4	Y5	Y6	Y7
Y1	0	0.91924	0.95376	0.90872	0.94968	0.92268
Y2	0.91924	0	0.87884	0.95992	0.87476	0.93596
Y4	0.95376	0.87884	0	0.94668	0.75904	0.86748
Y5	0.90872	0.95992	0.94668	0	0.93516	0.6024
Y6	0.94968	0.87476	0.75904	0.93516	0	0.90564
Y7	0.92268	0.93596	0.86748	0.6024	0.90564	0

Table S8.6. Alpha diversity index for ITS. Observed features and Shannon entropy per sample. P-value and H index based on Kruskal-Wallis (Cement Y5 Y6 Y7 (n=3), Rock Y1 Y2 Y4 (n=3)).

Sample	Observed_features	Shannon_entropy
Y1	145	2.61
Y2	211	4.46
Y4	387	6.27
Y5	144	1.83
Y6	184	4.94
Y7	168	4.03
H	1.19	0.43
P-value	0.275	0.513
